# Estimating the Serotype-Specific Association Between the Concentration of Vaccine-Induced Serum Antibodies and Protection Against Pneumococcal Colonization

**DOI:** 10.1093/infdis/jiaf106

**Published:** 2025-02-28

**Authors:** Anabelle Wong, Joshua L Warren, Laura Fitch, Stephanie Perniciaro, Ron Dagan, Daniel M Weinberger

**Affiliations:** Infectious Disease Epidemiology Group, Max Planck Institute for Infection Biology, Berlin, Germany; Institute of Public Health, Charité – Universitätsmedizin, Berlin, Germany; Department of Biostatistics, Yale School of Public Health, New Haven, Connecticut, USA; Public Health Modeling Unit, Yale School of Public Health, New Haven, Connecticut, USA; Public Health Modeling Unit, Yale School of Public Health, New Haven, Connecticut, USA; Department of Epidemiology of Microbial Diseases, Yale School of Public Health, New Haven, Connecticut, USA; Public Health Modeling Unit, Yale School of Public Health, New Haven, Connecticut, USA; Department of Epidemiology of Microbial Diseases, Yale School of Public Health, New Haven, Connecticut, USA; The Shraga Segal Department of Microbiology, Immunology, and Genetics, Faculty of Health Sciences, Ben Gurion University of the Negev, Beer Sheva, Israel; Public Health Modeling Unit, Yale School of Public Health, New Haven, Connecticut, USA; Department of Epidemiology of Microbial Diseases, Yale School of Public Health, New Haven, Connecticut, USA

**Keywords:** hierarchical Bayesian model, pneumococcal vaccine, nasopharyngeal colonization, immunogenicity, vaccine efficacy

## Abstract

**Background:**

Pneumococcal conjugate vaccines (PCVs) offer indirect protection by reducing pneumococcal colonization in the vaccinated children and thus transmission. As higher-valency PCVs may trigger a weaker immune response, it is important to understand how differences in immunogenicity between PCVs translate to effectiveness against colonization.

**Methods:**

We estimated the serotype-specific relationship between the concentration of vaccine-induced serum immunoglobulin G (IgG) and protection against colonization using a hierarchical Bayesian model with the longitudinal data from a randomized controlled trial. We then combined these estimates with the summary-level immunogenicity data (geometric mean concentrations and 95% confidence intervals) from head-to-head clinical trials comparing 13-valent versus 7-valent PCV (PCV13 vs PCV7), 15-valent PCV (PCV15) versus PCV13, and 20-valent PCV (PCV20) versus PCV13 to infer the relative effectiveness of higher-valency PCVs against colonization.

**Results:**

The hierarchical Bayesian model predicted that the risk of colonization increased as serum IgG decreased, and the association differed by serotype. Our approach estimated higher-valency PCVs to have lower vaccine effectiveness against colonization with some serotypes: 14 and 23F across comparisons; 4 when comparing PCV13 with PCV7 and PCV20 with PCV13; 5, 6A, 6B 7F, 19A, and 19F when comparing PCV15 and PCV20 with PCV13; and 1, 9V, and 18C when comparing PCV20 with PCV13.

**Conclusions:**

These findings suggest that while new PCVs might provide sufficient protection against disease, protection against transmission might be somewhat reduced for some serotypes. The overall impact should be evaluated in the local context, and further monitoring is critical to evaluate the impact of these changes in the coming years.

## Teaser

Combining summary-level immunogenicity data and a hierarchical Bayesian model trained on immunogenicity and colonization data showed that while new pneumococcal conjugate vaccines (PCVs) might sufficiently protect against disease, protection against transmission might be lower than existing PCVs for some serotypes.

The introduction of pneumococcal conjugate vaccines (PCVs) has substantially reduced the burden of invasive pneumococcal diseases (IPD) in children [1]. PCVs offer indirect protection to unvaccinated individuals by reducing pneumococcal colonization in children and, thus, the transmission from children to their contacts [2, 3]. This indirect protection also enhances the protection of vaccinated children.

The PCV-induced concentration of anticapsular immunoglobulin G (IgG) in serum is used as a correlate of protection against IPD. Immunogenicity measured in randomized controlled trials (RCTs) provides a basis of comparison between new PCVs and established PCVs and has been used as the basis for licensing new PCVs. Based on a meta-analysis [4], a serum IgG concentration of 0.35 μg/mL was set as the threshold of protection against IPD, mainly for the purpose of licensing new PCVs when efficacy studies are not feasible [5]. In reality, the threshold required for protection differs by serotype for IPD [6]. Protection against acquisition of nasopharyngeal colonization scales with higher antibody concentrations rather than having a fixed threshold associated with protection [7].

As higher-valency PCVs become available, a reduction in the strength of the immune response [8] may weaken protection against the acquisition of colonization for some serotypes. While the antibody concentration of the new PCVs is likely sufficient to protect against IPD [9], effectiveness against colonization may erode, as a stronger response is required to protect against colonization than diseases [7, 10].

There is an acute need to understand how differences in immunogenicity between PCVs translate to effectiveness against colonization. Previous work has assessed the association between postprimary antibody responses and risk of colonization in the first year of life [7]. However, transmission of pneumococcus is thought to be driven by preschool and school-aged children [11, 12]. Therefore, estimating the relationship between immunogenicity and risk of colonization in older children after receipt of the booster dose is important for understanding the differences in immunogenicity between PCVs for transmission. Estimates assessing the relationship between postbooster antibody responses and risk of colonization by serotype and risk group were unstable for many serotypes due to scarce events [10]. Reanalyzing these data using advanced analytical methods can help stabilize the estimated serotype-specific relationship between serum antibody levels and protection against colonization [13].

**Figure 1.**
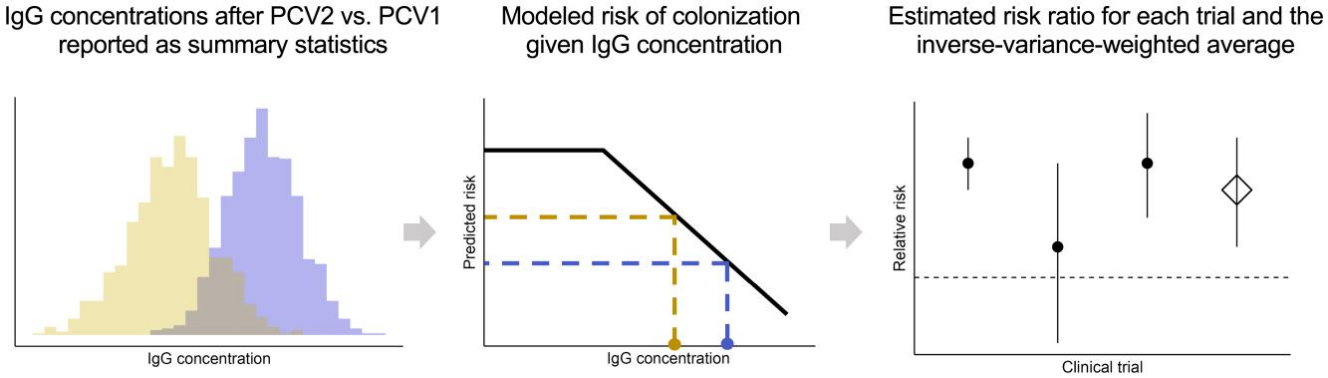
Schematic showing the modeling workflow. *Left panel,* Summary-level serotype-specific immunogenicity data (means of immunoglobulin G [IgG] concentration with 95% confidence intervals) from head-to-head clinical trials comparing a higher-valency pneumococcal conjugate vaccine (PCV) (PCV2) with an older, lower-valency PCV (PCV1) are publicly available. *Middle panel,* Combining these data with a hierarchical Bayesian model fitted to the longitudinal data on post-PCV immunogenicity and colonization acquisition from a randomized controlled trial [10, 14] gives the relative risk of colonization for each head-to-head trial. *Right panel,* Estimated relative risk of colonization for all included head-to-head trials for a specific comparison (eg, 13-valent vs 7-valent PCV) were pooled using inverse-variance weighting.

In the current study, we estimated the relationship between serum IgG and risk of colonization by serotype by reanalyzing data from a RCT in Israel using a hierarchical Bayesian model. We combined these estimates with summary-level immunogenicity data from head-to-head clinical trials to infer the relative effectiveness of new PCVs against colonization (Figure 1).

## METHODS

### Data

We used 2 sets of data in this study: (1) longitudinal data on post-PCV immunogenicity and colonization from a RCT and (2) summary-level immunogenicity data from head-to-head clinical trials. The first dataset (clinical trial registration NCT00508742) was described elsewhere [10, 14]. The serotype-specific IgG concentration was measured using standardized enzyme-linked immunosorbent assay (ELISA) 1 month after children received their booster dose (ie, at age 13 months) with either 7-valent PCV (PCV7) or 13-valent PCV (PCV13). The ethnicity of the children (Bedouin or Jewish) was recorded. Nasopharyngeal swab samples were collected and cultured at 2, 4, 6, 7, 12, 13, 18, and 24 months of age to detect acquisition of colonization. Positive samples were serotyped using Quellung reaction. Pneumococcal acquisition was recorded as a binary outcome for each of the 13 serotypes included in PCV13 (4, 6B, 9V, 14, 18C, 19F, 23F, 1, 3, 5, 6A, 7F, and 19A). For postbooster IgG, we examined colonization prior to 13 months, and if a participant was found to be colonized by a PCV13 serotype, subsequent observations for that serotype were censored (Supplementary Appendix 1). In addition to postbooster data, we analyzed the association between serotype-specific IgG concentration 1 month after the primary series (ie, at age 7 months) and the risk of postbooster colonization (Supplementary Figure 10).

The second dataset came from head-to-head trials comparing PCV13 versus PCV7, 15-valent PCV (PCV15) versus PCV13, and 20-valent PCV (PCV20) versus PCV13. A scoping review provided a list of studies evaluating the immunogenicity of PCVs [15]. Head-to-head clinical trials with results including postprimary or postbooster IgG geometric mean concentrations (GMCs) were selected (Supplementary Figures 2–4). Summary-level immunogenicity data (GMCs and 95% confidence intervals [CIs]) were extracted from WISSPAR.com and ClinicalTrials.gov.

The analyses of these deidentified data were exempted from review by the Yale University institutional review boards (protocol no. 2000038285).

### Risk of Colonization and Serum IgG Concentration

Pneumococcal vaccines affect colonization by preventing the acquisition of pneumococcus rather than shortening the duration of colonization. Given the longitudinal data with measurements at just 3 time points, we cannot assess acquisition rates. Therefore, we assessed the risk of colonization as a function of antibody concentration at 13 months. To model this, we assumed that, for a given serotype, the risk of colonization decreased with increasing serum IgG concentration, following a log-log linear relationship. We also assumed that there was a minimum amount of antibody (ie, the change point) needed before any reduction in risk was observed. We developed a hierarchical Bayesian change point model to allow for the sharing of information across different serotypes during model fitting, resulting in more robust estimates of serotype-specific relationships.

The model was fitted to the longitudinal data on post-PCV immunogenicity and colonization [10, 14] to estimate serotype-specific intercepts, change points, slopes, and ethnicity-specific intercepts. Details of the model are given in Supplementary Appendix 2. As a sensitivity analysis, we tested a less informative prior for $\mu_{cp}$, the mean of the serotype-specific change point parameter (Supplementary Appendix 3). We also tested a simpler linear model (without change point) including random effects for ethnic groups (Supplementary Appendix 4). The estimates of the relationship between IgG concentration and risk of colonization for ethnic groups were not notably different in preliminary analyses (Supplementary Figure 10); therefore, we accounted only for the baseline colonization risk difference by including the ethnicity-specific intercepts. This assumes that the relative reductions in risk of colonization achieved with changes in antibody concentration were the same between ethnic groups, but the baseline risk of colonization differed.

### Relative Vaccine Efficacy

We extracted the GMCs of IgG (with 95% CIs) measured with ELISA 1 month after the booster from head-to-head trials identified in a review comparing several PCVs [15]. For trials involving PCV15, only GMCs measured by electrochemiluminescence were reported. Therefore, we converted electrochemiluminescence-measured GMC to ELISA-measured GMC [16, 17] (Supplementary Appendix 5).

Using the fitted hierarchical Bayesian model, we predicted the risk of colonization with a given serotype for each reported GMC. For the head-to-head comparison in each trial, we divided the predicted risk from a higher-valency PCV by the predicted risk of a lower-valency PCV to obtain the relative risk (RR). We pooled the estimated RR across trials using inverse-variance weights (Supplementary Appendix 6). The uncertainty of the estimates came from the posterior samples of the hierarchical Bayesian model and the reported variance of the GMC. With the RR of colonization, we obtained the relative vaccine efficacy (VE) as 1 − RR = relative VE; that is, the higher the RR, the lower the relative VE. This can also be interpreted in terms of absolute VE. For example, assuming that PCV7 confers 60% (RR, 0.4) protection against colonization by a serotype [18, 19], an estimated RR of 1.10 when comparing PCV13 with PCV7 can be interpreted as 10% reduction in the absolute VE for the higher-valency PCV, meaning that PCV13 has an absolute VE of 56% (1 − 0.4 ⋅ 1.1). If the RR for PCV20 versus PCV13 was 1.2, we would extend this by calculating the overall VE as 47% (1 − 0.4 ⋅ 1.1 ⋅ 1.2).

## RESULTS

### Overview of Data

After excluding baseline colonization episodes and censoring colonization episodes of the same serotype following the first event, there were 28192 measurements from 2345 samples, obtained from 896 children [10, 14] (Supplementary Table 1). Among the included observations, there were 220 newly acquired colonizations. After identifying registered head-to-head clinical trials for PCV13v7 (7 of 19), PCV15v13 (9 of 19), and PCV20v13 (4 of 13), we extracted the GMCs with 95% CIs from completed studies for postprimary and postbooster IgG against PCV13 serotypes (Supplementary Figures 2–4).

### Relationship Between Risk of Colonization and Antibody Concentration

**Figure 2.**
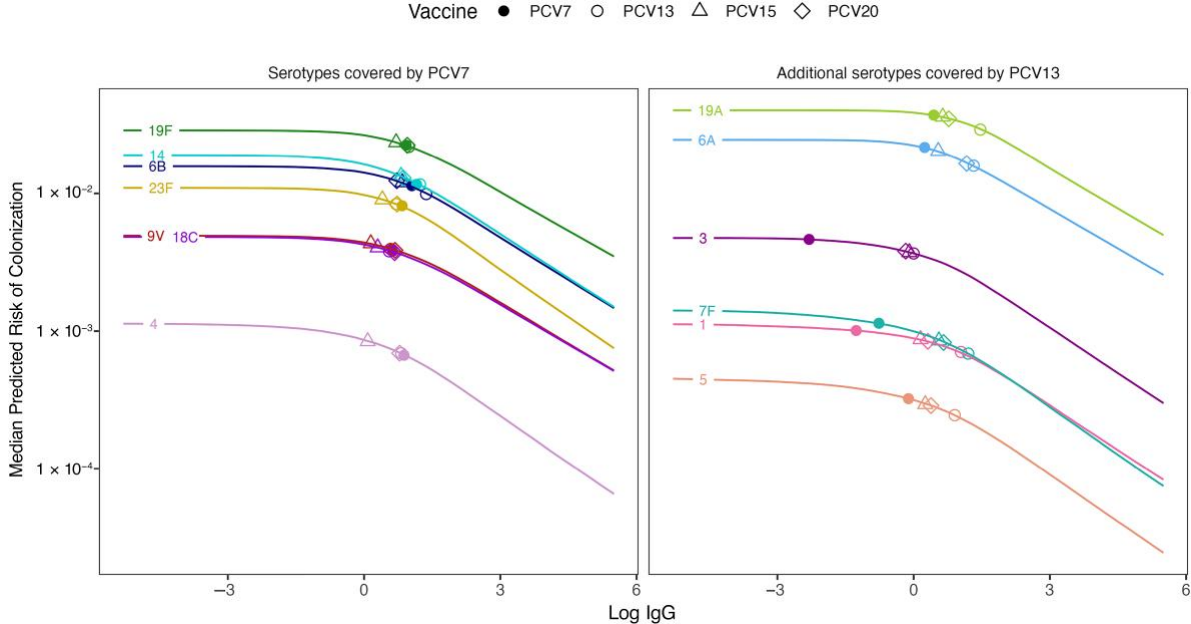
Predicted probability of colonization by antibody concentration for each serotype. The median predicted risk of colonization from the hierarchical Bayesian model for each serotype is plotted against the concentration of serum antibodies. *Left panel,* Serotypes covered by 7-valent pneumococcal conjugate vaccine (PCV) (PCV7; serotypes 4, 6B, 9V, 14, 18C, 19F, and 23F). *Right panel,* Additional serotypes covered by 13-valent PCV (PCV13; serotypes 1, 3, 5, 6A, 7F, and 19A). Points indicate antibody concentrations achieved by the booster dose of PCV7 (*filled circles*), PCV13 (*open circles*), 15-valent PCV (PCV15; *open triangles*), and 20-valent PCV (PCV20; *open diamonds*) as an inverse-variance-weighted average of the immunoglobulin G (IgG) geometric mean concentrations reported by the head-to-head clinical trials. The y-axis is displayed in the log_10_ scale.

When fitted to the data on post-PCV immunogenicity and colonization [10, 14], the model estimated a decline in risk of colonization with increasing serum IgG concentration (Figure 2). For most serotypes (6B, 9V, 14, 18C, 19F, 23F, 3, 6A, 19A), the decline in risk only became more apparent above certain serum IgG concentration, suggesting a potential minimal concentration necessary for protection against colonization; for other serotypes (4, 1, 5, and 7F), the predicted risk declined more consistently with increasing serum IgG concentration across the range of observed values (Supplementary Figures 5 and 6). Notably, the latter serotypes had few data points (Supplementary Table 1). Although the change points appeared slightly more subtle when using a less informative prior for the hyperparameter $\mu_{cp}$ in the sensitivity analysis (Supplementary Figure 7), the overall shape of the risk curves remained similar (Supplementary Figure 8). The baseline risk of colonization was higher in the Bedouin group than in the Jewish groups (Supplementary Figure 5) and differed by serotype (Figure 2).

The linear model showed no evidence for differential relative reductions in risk of colonization between the ethnic groups (Supplementary Figure 10). This model predicted a higher-valency PCV to be associated with a higher risk of colonization with most serotypes for both Bedouin and Jewish children considering the postbooster but not the postprimary IgG concentration (Supplementary Figure 10), suggesting that such effects may only become apparent after the booster dose. In general, the estimated RRs of colonization from the change point model were similar to that from the linear model but with larger uncertainty intervals (Supplementary Figure 11). The inference of RR of colonization focuses on the postbooster results from the change point model.

### RR of Colonization With the 7 Shared Serotypes

**Figure 3.**
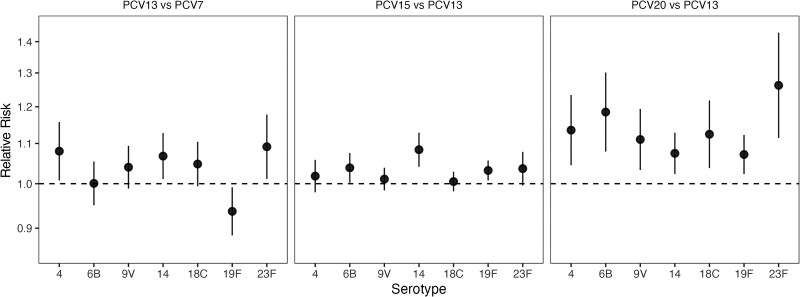
Relative risk (RR) of colonization with the 7 shared serotypes for 13-valent pneumococcal conjugate vaccine (PCV) (PCV13) versus 7-valent PCV (PCV7), 15-valent PCV (PCV15) versus PCV13, and 20-valent PCV (PCV20) versus PCV13. The estimated RRs of colonization with the 7 serotypes commonly covered by PCV7, PCV13, PCV15, and PCV20 (4, 6B, 9V, 14, 18C, 19F, and 23F), comparing PCV13 with PCV7 (*left panel*), PCV15 with PCV13 (*middle panel*), and PCV20 with PCV13 (*right panel*), are displayed as means (*points*) with 95% credible intervals (*error bars*). The y-axis is displayed in the log_10_ scale. RRs above 1 (ie, above the dashed lines) represent a higher risk of colonization with use of the higher-valency PCVs.

Across the 3 head-to-head comparisons, the risk of colonization with serotype 14 was 7%–8% higher with use of a higher-valency PCV compared with a lower-valency PCV (RR [95% credible interval (CrI)], 1.07 [1.01–1.13] for PCV13v7, 1.08 [1.04–1.13] for PCV15v13, and 1.07 [1.02–1.13] for PCV20v13; Figure 3). For interpretation, we assumed that the vaccine effectiveness against colonization for PCV7 was 60% [18, 19]. Hence, the reduction in immunogenicity would result in a VE against colonization of 57% for PCV13 and 54% for PCV15 and PCV20 (Supplementary Figure 15). For serotype 23F, the risk of colonization was 4%–26% higher when using a higher-valency PCV compared with a lower-valency PCV (RR [95% CrI], 1.09 [1.01–1.18] for PCV13v7, 1.04 [1.00–1.08] for PCV15v13, and 1.26 [1.11–1.43] for PCV20v13), with a high degree of uncertainty for PCV13 versus PCV7 and PCV20 versus PCV13. This would represent a VE against colonization of 56% for PCV13, 55% for PCV15, and 45% for PCV20 (Supplementary Figure 15).

For serotype 6B, the risk of colonization was higher when using PCV15 versus PCV13 (RR [95% CrI], 1.03 [1.00–1.08]) and when using PCV20 versus PCV13 (1.18 [1.08–1.30]), resulting in 59% effectiveness for PCV15% and 53% for PCV20 (Supplementary Figure 15). There was little evidence for a difference in risk for PCV13 versus PCV7 (RR [95% CrI], 1.00 [.95–1.05]). The risk for serotype 4 was higher when using PCV13 versus PCV7 (RR [95% CrI], 1.08 [1.01–1.16]) and when using PCV20 versus PCV13 (1.14 [1.04–1.23]) but not when using PCV15 versus PCV13 (1.02 [.98–1.06]).

For other serotypes, the differences in risk of colonization for PCV13 versus PCV7 and PCV15 versus PCV13 were not notably different, except for serotype 19F, for which the risk was lower with PCV13 versus PCV7 (RR [95% CrI], 0.94 [.88–.99]) and slightly higher with PCV15 versus PCV13 (1.03 [1.01–1.06]; Figure 3). Comparing PCV20 versus PCV13, the risks of colonization with all serotypes were higher when using PCV20, though the uncertainty was higher with some (RR [95% CrI], 1.11 [1.03–1.19] for 9V and 1.12 [1.04–1.22] for 18C) than with others (1.07 [1.02–1.12] for 19F).

### RR of Colonization With 6 Additional PCV13 Serotypes

**Figure 4.**
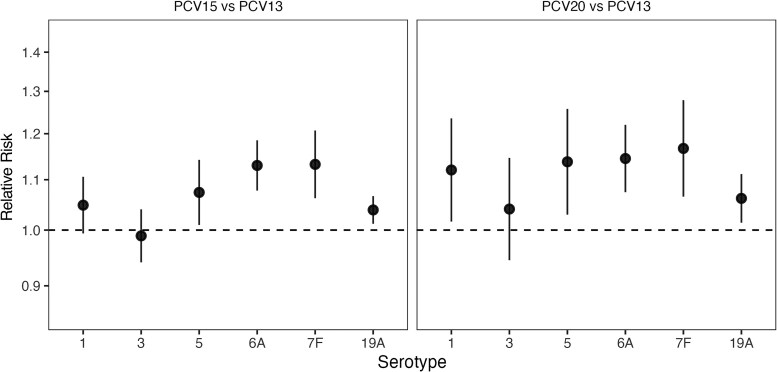
Relative risk (RR) of colonization with 6 additional shared serotypes for 15-valent pneumococcal conjugate vaccine (PCV) (PCV15) versus 13-valent PCV (PCV13) and 20-valent PCV (PCV20) versus PCV13. The estimated RRs of colonization with the 6 additional serotypes commonly covered by PCV13, PCV15, and PCV20 but not by 7-valent PCV (PCV7; serotypes 1, 3, 5, 6A, 7F, 19A), comparing PCV15 with PCV13 (*left panel*) and PCV20 with PCV13 (*right panel*), are displayed as means (*points*) with 95% credible intervals (*error bars*). The y-axis is displayed in the log_10_ scale. RRs above 1 (ie, above the dashed lines) represent a higher risk of colonization with use of the higher-valency PCVs.

For both PCV15 versus PCV13 and PCV20 versus PCV13, the risks of colonization with serotypes 5, 6A, 7F, and 19A were higher when using a higher-valency PCV. Comparing PCV15 and PCV13, the risks of colonization with serotypes 6A and with 7F were both 13% higher when using PCV15 (RR [95% CrI], 1.13 [1.08–1.18] for 6A and 1.13 [1.06–1.21] for 7F; Figure 4). For serotype 6A, there has been cross-reactivity described with serotype 6B, so these results should be interpreted relative to the VE obtained from cross-reactivity with 6B in PCV7 or PCV13.

The higher risks of colonization with these serotypes were more pronounced when comparing PCV20 and PCV13, but with higher uncertainty (RR [95% CrI], 1.14 [1.07–1.22] for 6A and 1.17 [1.07–1.28] for 7F). For serotypes 19A and 5, the risks of colonization were estimated to be 4%–6% and 7%–14% higher, respectively, when using PCV20 in both comparisons, with higher uncertainty for serotype 5 (RR [95% CrI], 1.07 [1.01–1.14] for PCV15v13 and 1.14 [1.03–1.26] for PCV20v13) than for serotype 19A (1.04 [1.01–1.07] for PCV15v13 and 1.06 [1.01–1.11] for PCV20v13). While the risk of colonization with serotype 1 was higher with PCV20 than with PCV13 (RR [95% CrI], 1.12 [1.02–1.24]), the risk of colonization with serotype 3 was not notably different for either PCV15 versus PCV13 or PCV20 versus PCV13 in the main analysis. When using a less informative prior for the hyperparameter $\mu_{cp}$ in the sensitivity analysis, the protection against colonization with serotype 3 was estimated to be better for PCV15 than for PCV13 and worse for PCV20 than for PCV13 (Supplementary Figure 9).

## DISCUSSION

Using a hierarchical Bayesian model, we reanalyzed longitudinal data on post-PCV immunogenicity and colonization from a RCT [10, 14] to estimate the serotype-specific relationship between the concentration of serum IgG and the risk of colonization. These findings suggest that for PCV15 and PCV20 there might be a modest drop in effectiveness against colonization. Effectiveness against colonization overall is still moderate, and further work is needed to determine the implications of changes in immunogenicity on colonization of PCV-targeted serotypes. In a population with a well-established PCV program with high booster coverage, the modest differences in risk of colonization described here may not have a population-level impact on carriage and transmission. Nonetheless, these data cannot rule out the tradeoffs between maintaining population-level protection and increasing the number of serotypes covered when comparing different PCVs.

Compared with a previous study using these data [14], the statistical approach used here has the advantage of stabilizing the estimates for serotypes with sparse colonization events [13]. Other work estimated the relationship between immunogenicity and seroincidence of pneumococcus [7]. Those studies focused on the period after the primary series but before the booster dose [7, 20]. While there is a correlation between postprimary and postbooster responses, differences among serotypes are generally less pronounced after the booster dose. Since controlling carriage in older children is key for maintaining indirect protection, understanding the immune response during this postbooster period is critical.

Our model predicted that the risk of colonization increased as serum IgG decreased, and the association differed by serotype (Figure 2) but not by ethnicity (Supplementary Figure 10), as described elsewhere [14]. Furthermore, the results from our model suggested that a higher concentration of serum IgG may be necessary to protect against colonization with serotypes 6B, 14, 19F, and 23F, as observed elsewhere [7], and with 3 additional serotypes—3, 6A, and 19A—in our study. These results may explain the limited indirect effects of PCVs against some serotypes, such as serotype 3, 4, 19A, and 19F in Germany [21], although future studies are required to translate the protection against colonization in children to the changes in risk in other age groups.

Combining the risk curves from our model with the immunogenicity data from clinical trials, we inferred the relative VE for new PCVs. This relative VE reflects protection against colonization in vaccinated children. Our approach estimated lower VE for serotypes 4, 14, 5, 6A, and 7F when comparing PCV15 versus PCV13 and PCV20 versus PCV13. In addition, PCV20 was estimated to have lower VE also against colonization with serotypes 6B and 23F. Therefore, it will be essential to remain vigilant in monitoring serotype-specific carriage and disease when switching to higher-valency PCVs.

Our study has several limitations. First, we base our model on serum IgG, which provides a correlate of protection but does not reflect all mechanisms of protection, such as T-cell response [22] and mucosal immunity [23, 24], which are difficult to measure. Second, the longitudinal data used for fitting the hierarchical Bayesian model came from a single location, which limits the generalizability of our model; nevertheless, estimates for 2 ethnic groups were compared using the linear model, and we saw no evidence for differential protection against colonization by ethnicity (Supplementary Figure 10). Data from wider populations are necessary to confirm this. Third, our model relies on data from RCTs, which represent scenarios of healthy infants with high immunization schedule compliance. It would be helpful to validate our model against real-world data when the newly approved higher-valency PCVs become more widely used. Finally, although a hierarchical model can reduce noise and stabilize the estimates by sharing information across serotypes, it may introduce bias for serotypes with unique behaviors, for example, serotype 3. A hybrid model that incorporates a hierarchical model for similar serotypes with the flexibility to model unique serotypes independently may help address this limitation.

Using a novel approach to compare higher-valency PCVs, our study offers directly interpretable estimates of the RR of colonization using different PCVs. These results highlight the importance of continuous monitoring of the distribution of serotypes in carriage and disease in the era of higher-valency PCVs. There would likely be a lag of a few years before any population-level impacts of reduced immunogenicity would become apparent. In addition to surveillance, future research efforts are indispensable in evaluating the impact of new vaccines. Primary data on immunogenicity and colonization should be collated from available clinical trials. As more evidence based on various end points (IgG, memory B cells, colonization, and disease) from reduced-dose (1 + 1 and 0 + 1) schedules [25–27] and fractional dose regimens becomes available [28], our approach may also help answer similar questions about the relative effectiveness of various dosing regimens, with the prospect of considering duration of protection.

In conclusion, these analyses suggest that while new PCVs might provide sufficient protection against severe disease, protection against transmission might be somewhat reduced for some serotypes. This could have effects on undervaccinated age groups and other high-risk individuals. The overall impact should be evaluated in the local context, taking into account the proportion of fully vaccinated individuals. As such, further monitoring is critical to evaluate the impact of these changes in the coming years.

## Supplementary Material

jiaf106_Supplementary_Data

## Supplementary Data

Supplementary materials are available at *The Journal of Infectious Diseases* online (http://jid.oxfordjournals.org/). Supplementary materials consist of data provided by the author that are published to benefit the reader. The posted materials are not copyedited. The contents of all supplementary data are the sole responsibility of the authors. Questions or messages regarding errors should be addressed to the author.

## Notes

***Author contributions*.** Study conception, supervision of data analyses, project administration, and provision of resources: D. M. W. Design and coding of models: A. W., J. L. W., and D. M. W. Literature review: L. F. and S. P. Curation and cleaning of data: L. F., S. P., and R. D.

Data analysis and writing of first manuscript draft: A. W. Contributions to methodology, interpretation of results, and revision of manuscript: A. W., J. L. W., L. F., S. P., R. D., and D. M. W. All authors approved the final version of the submitted manuscript.

***Acknowledgments.*** A. W.’s research stay was supported by the Domenech Lab at the Max Planck Institute for Infection Biology. We thank Jessica Weaver, Josiah Thomas, and Maurice Ahsman for advice on the use of the conversion of electrochemiluminescence to ELISA unit based on their previous publication.

***Code and data availability***. Analysis code and data from the scoping review are available online (https://github.com/PneumococcalCapsules/PCV_Ab_colonization).

***Financial support.*** This work was partially supported by the Gates Foundation (grant INV-065870).
